# Salt Spray Aging Behavior and Life Prediction of Thermoplastic Carbon Fiber-Reinforced Poly(methyl methacrylate) Composites

**DOI:** 10.3390/ma19183968

**Published:** 2026-09-18

**Authors:** Xiaofeng Guo, Shiyun Li, Guangtao Li, Yifan Wang, Jianmin Zhang

**Affiliations:** 1 School of Intelligent Mechatronics Engineering, Zhongyuan University of Technology, Zhengzhou 450007, China; yunyunxxy@163.com (S.L.); lgtdqly@163.com (G.L.); wiyifanwyf@163.com (Y.W.); 2 Longzihu New Energy Laboratory, Zhengzhou Institute of Emerging Industrial Technology, Zhengzhou 450000, China; 3 Beijing Key Laboratory of Ionic Liquids Clean Process, Institute of Process Engineering, Chinese Academy of Sciences, Beijing 100190, China

**Keywords:** poly(methyl methacrylate), carbon fiber fabric, salt spray aging, mechanical properties, life prediction

## Abstract

To investigate the salt spray durability of recyclable carbon fiber-reinforced poly(methyl methacrylate) (PMMA) composites (CF/PMMA), laminates were fabricated via vacuum-assisted resin transfer molding (VARTM), and salt spray accelerated aging tests were conducted. Water uptake tests, mechanical tests (0°/90° tensile, flexural, short-beam shear), and SEM micro-characterization of surface and fracture morphologies were performed to compare the performance degradation and interfacial deterioration of the two systems under salt spray conditions, and the residual strength was predicted using the median strength aging equation model. The results show that unaged CF/PMMA exhibits higher initial mechanical properties than carbon fiber-reinforced epoxy composites (CF/Epoxy) and lower equilibrium water uptake due to fewer polar groups in the salt spray aging process. After 960 h of aging, CF/PMMA retains better mechanical properties (retention rates: 68.19%, 78.94%, 84.03%, and 76.28% in 0° tensile, 90° tensile, flexural, and shear strengths, respectively) than CF/Epoxy. Based on the life prediction model, under the artificial accelerated salt spray aging test condition and with 50% strength retention as the failure threshold, the predicted tensile and shear service lives are 99.05 days and 389.06 days, respectively. This study elucidates the salt spray aging mechanism of PMMA-based thermoplastic carbon fiber composites, providing a reference for their marine engineering applications.

## 1. Introduction

Carbon fiber (CF)-reinforced composite is a class of structural composite material consisting of a resin matrix and carbon fiber reinforcement. Featuring high specific strength, high modulus and excellent fatigue resistance, it has been widely adopted in aerospace, wind-power equipment, automobile manufacturing, ocean engineering and pipeline transportation. It serves as a critical material for structural light weighting, service life improvement and energy-consumption reduction [1,2,3,4]. In ocean engineering applications, composite components operate continuously in marine atmospheric environments characterized by high salinity and humidity. Sustained salt spray exposure induces multi-scale damage including resin matrix degradation, weakened interfacial bonding and fiber performance deterioration [5,6], which eventually degrades mechanical properties and reduces the durability and safety of composite structures [7,8,9]. Accordingly, the systematic investigation of the mechanical property evolution and degradation mechanisms of carbon fiber-reinforced composites under salt spray aging is of great theoretical value and engineering significance for the life prediction, optimized anti-corrosion design and practical deployment of marine composite components [10,11].

Carbon fiber-reinforced polymer (CFRP) can be mainly divided into two categories based on matrix: thermoset and thermoplastic. Epoxy matrix composites, as an example of thermoset composites, possess mature manufacturing processes and superior interfacial adhesion and have long dominated the composite market. Sufficient fundamental research and engineering practices have been established for epoxy-based composites [12,13]. Nevertheless, cross-linked networks formed after thermoset curing prevent re-melting and reprocessing. Drawbacks, including poor recyclability and insufficient fracture toughness, limit their adaptability to modern requirements for green manufacturing, high-efficiency production and sustainable development [14,15,16]. Pender et al. [17] performed a life-cycle assessment and demonstrated that the landfill and incineration of carbon fiber composites fail to achieve effective carbon reduction and instead continuously emit greenhouse gases and toxic organic pollutants. Fette et al. [18] reviewed chemical-recycling routes for thermoset composites and pointed out that solvent depolymerization and catalytic degradation require harsh, high-temperature, corrosive conditions, which readily damage fiber micro-morphology and degrade fiber performance, leading to the low recovery efficiency of intact carbon fiber fabrics. Kuila et al. [16] further illustrated that irreversible cross-linked networks in cured thermoset resins constitute the core bottleneck for material recycling. Mechanical grinding and pyrolysis only produce low-grade short fibers, which cannot be reused to fabricate high-performance load-bearing composites.

By contrast, thermoplastic matrix composites such as polypropylene-based [19] and polyamide-based [20,21] systems exhibit prominent advantages including recyclability, superior impact toughness, short molding cycles and a long shelf life and have become major research hotspots in composite materials [22,23]. Poly(methyl methacrylate) (PMMA) is a typical amorphous thermoplastic resin with low density, favorable mechanical performance, high optical transmittance and good chemical resistance [24,25,26,27]. Recently, Zhang et al. [28] developed a novel PMMA-based thermoplastic resin, namely, PMMA/MMA binary liquid resin. This design takes advantage of PMMA’s solubility in MMA monomer, and precise viscosity tuning is achieved by adjusting the PMMA/MMA mass ratio. Wang et al. [29] systematically optimized vacuum infusion and conventional hot-press molding processes for carbon fiber/PMMA prepregs and fabricated composite panels with low porosity and excellent tensile properties. Zheng et al. [30] modified the PMMA/MMA liquid resin system and manufactured high-performance CF/PMMA composites via vacuum-assisted resin transfer molding (VARTM) under low-temperature curing. Experimental results reported by Gao [26] and Ren et al. [31] confirmed that PMMA matrix composites possess obvious advantages in low-temperature stability and recyclability.

Regarding the property degradation of CFRP in salt spray environments, Wang et al. [32] carried out durability tests on CFRP–steel joints exposed to neutral salt spray and established quantitative correlations between salt spray aging duration and residual joint strength. Their results revealed that salt spray media diffuse along fiber–matrix interfaces, gradually deteriorate interfacial adhesion and, ultimately, reduce joint load-bearing capacity significantly. Li et al. [33] explored the influences of temperature and salinity on the static and dynamic mechanical behaviors of CFRP under seawater aging. High temperature and high salinity accelerate water diffusion inside composites, aggravating matrix hydrolysis and interfacial degradation. After seven-month immersion in 30 °C seawater, the maximum tensile strength loss in the 0° fiber direction reached 11.5%. Fiore et al. [34] investigated the salt spray aging of hybrid composite–metal riveted joints for automobiles. Salt spray corrosion preferentially attacks interfacial regions, triggers metallic-substrate corrosion and composite interlayer delamination, and continuously decreases joint shear strength. Cunha et al. [35] performed water immersion and ozone aging tests on unidirectional carbon–epoxy laminates. They found that water penetration aggravates interfacial degradation, and high temperature amplifies the tensile strength reduction induced by water uptake. Berges et al. [36] utilized quasi-static, fatigue and vibration tests to characterize hygroscopic effects on unidirectional flax–epoxy composites. Their results showed that moisture absorption reduced elastic modulus and increased damping, while monotonic tensile strength remained unchanged. Liu et al. [37] conducted accelerated salt spray aging experiments and found that glass fiber composites gained mass via water absorption with prolonged aging time, accompanied by severe mechanical property decay, especially in the early aging stage. Wang et al. [38] investigated the degradation behaviors of carbon fiber hybrid pultruded epoxy composites under salt spray and hygrothermal conditions. In both environments, only physical water absorption occurs alongside the formation of fiber–matrix interfacial microcracks, which gives rise to a gradual and eventually saturated decline in flexural and impact performance.

A review of the existing literature reveals that most studies on the mechanical properties of fiber-reinforced composites and the behavior of salt spray aging concentrate on epoxy-based counterparts. The mechanical property, degradation behaviors and intrinsic mechanisms of carbon fiber-reinforced PMMA composites upon salt spray exposure have not been well studied.

Targeting this research gap, this work selects CF/PMMA composite as the research subject and CF/Epoxy composite with identical fabric architecture as the reference. Salt spray aging tests were performed following the ASTM B117 standard. Water absorption measurements and mechanical tests were adopted to characterize the mechanical property evolution of both composite systems during salt spray aging. Scanning electron microscopy (SEM) was employed to reveal salt spray-induced degradation mechanisms from a micro-morphological perspective, and the residual strength was predicted using the median strength aging equation model. This study provides experimental data and references for the structural design, life assessment and engineering applications of such thermoplastic composites in coastal and marine engineering scenarios.

## 2. Materials and Methods

### 2.1. Materials

Poly(methyl methacrylate) (PMMA) powder with a number-average molecular weight (Mn) of 1 × 10^5^ g/mol was purchased from Dongguan Hongxing New Material Co., Ltd. (Dongguan, China); Methyl methacrylate (MMA, 99.9%) and N,N-dimethylaniline (DMA) were obtained from Shandong Keyuan Pharmaceutical Co., Ltd. (Jinan, China); Benzoyl peroxide (BPO, 98%) was supplied by Shanghai Macklin Biochemical Co., Ltd. (Shanghai, China); Unidirectional carbon-fabric (Model: HF-QR-8.2-17) was provided by Changzhou Hongfa Advanced Materials Co., Ltd. (Changzhou, China). Epoxy resin (Model: LT-5028A) and its corresponding curing agent (Model: LT-5028B) were purchased from Wells Advanced Materials (Shanghai) Co., Ltd. (Shanghai, China); Sodium chloride (NaCl) was obtained from Tianjin Hengxing Chemical Reagent Co., Ltd. (Tianjin, China).

**Figure 1:**
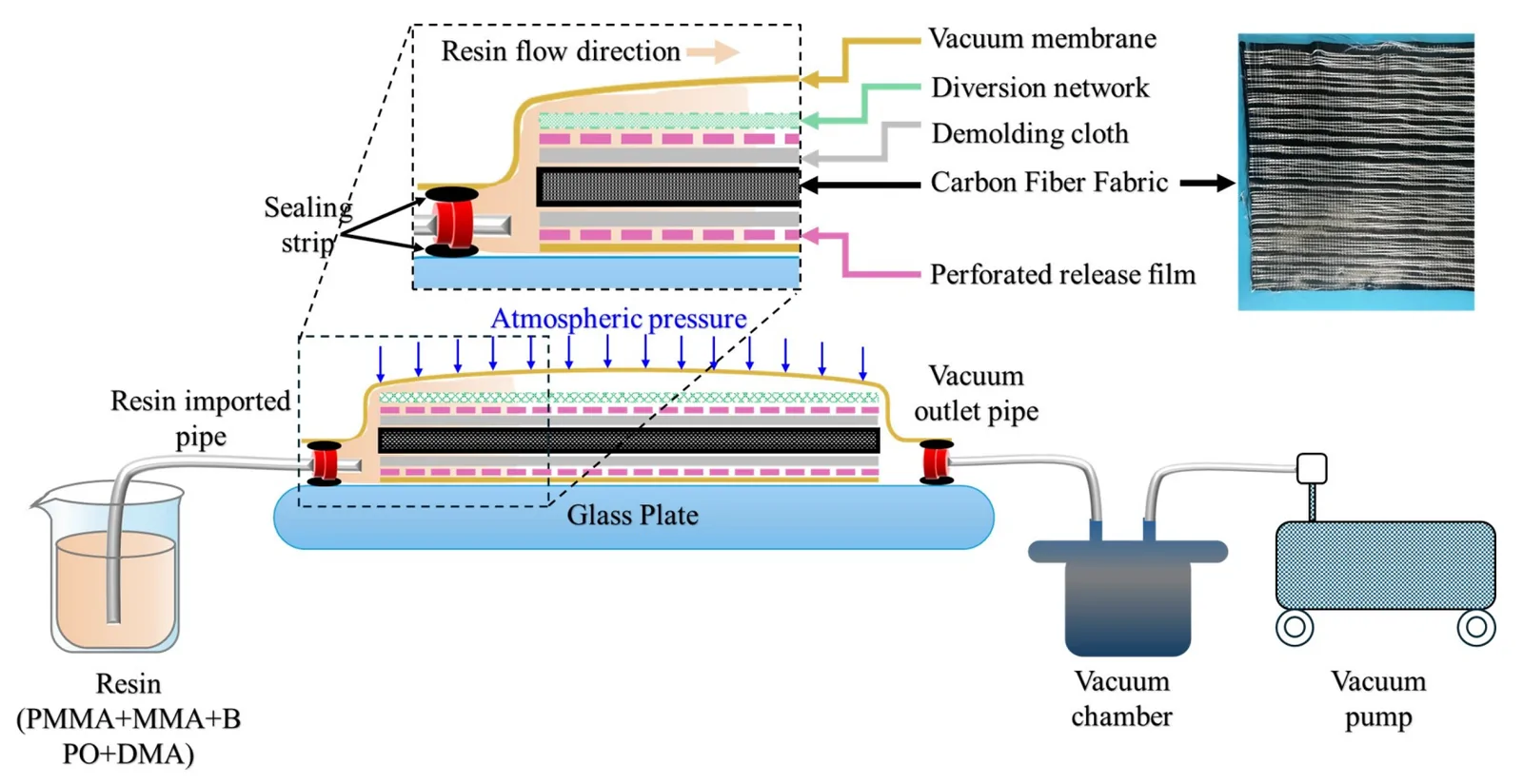
Schematic of the vacuum-assisted resin infusion molding process for CF/PMMA composite laminates.

The schematic of the vacuum-assisted resin infusion molding process for carbon fiber/polymethyl methacrylate (CF/PMMA) composite laminates is shown in Figure 1.

### 2.2. Preparation of CF Composites

Figure 1 illustrates the vacuum-assisted resin impregnation process for CF/PMMA composite laminates. First, the carbon fiber fabric was fully pre-dried in an oven at 80 °C for 4 h. Subsequently, the dried carbon fiber preform was placed into a mold and held under a vacuum pressure of 1 MPa for 20 min. Second, the PMMA resin was prepared: 806.5 g of MMA was mixed with 193.5 g of PMMA (M_n_ = 1 × 10^5^), and the mixture was stirred in an oscillator at 45 °C and 200 rpm for 12 h until PMMA was completely dissolved in MMA; this obtained a PMMA+MMA binary resin system with a mass fraction of 24 wt%. Then, the initiators BPO and DMA were added in appropriate proportions, and the mixture was stirred for 5 min. The mass ratio of MMA to the initiators was MMA: BPO: DMA = 100:1.2:1 (The selection of this ratio was based on previous research conducted by our group [29,30], which demonstrated their excellent overall performance.) Vacuum-assisted resin transfer molding (VARTM) was adopted, and the mixed resin solution was injected into the mold at a pressure of 0.095 MPa. The composite was cured at 35 °C for 12 h, followed by post-curing in an oven at 80 °C for 4 h, to ultimately obtain the CF/PMMA composite.

Epoxy resin LT-5028A and curing agent LT-5028B were mixed at a mass ratio of 100:30. The CF/epoxy composite was also prepared via the VARTM method, with initial curing at 50 °C for 6 h followed by post-curing at 70 °C for 4 h, which finally yielded the CF/Epoxy composite. The measured mass fractions (W_f_) of the carbon fiber fabric were 74.11 ± 1.5% for CF/PMMA and 72.09 ± 1.5% for CF/Epoxy.

### 2.3. Artificial Accelerated Salt Spray Aging Test

In this test, the neutral salt spray (NSS) accelerated aging test was carried out in accordance with the ASTM B117 standard [39]. Referring to the principle of standard test gradient design, four aging durations, namely, 0 h (unaged control group), 240 h, 480 h, and 960 h, were selected to analyze the performance evolution of the material at different aging stages [34].

**Figure 2:**
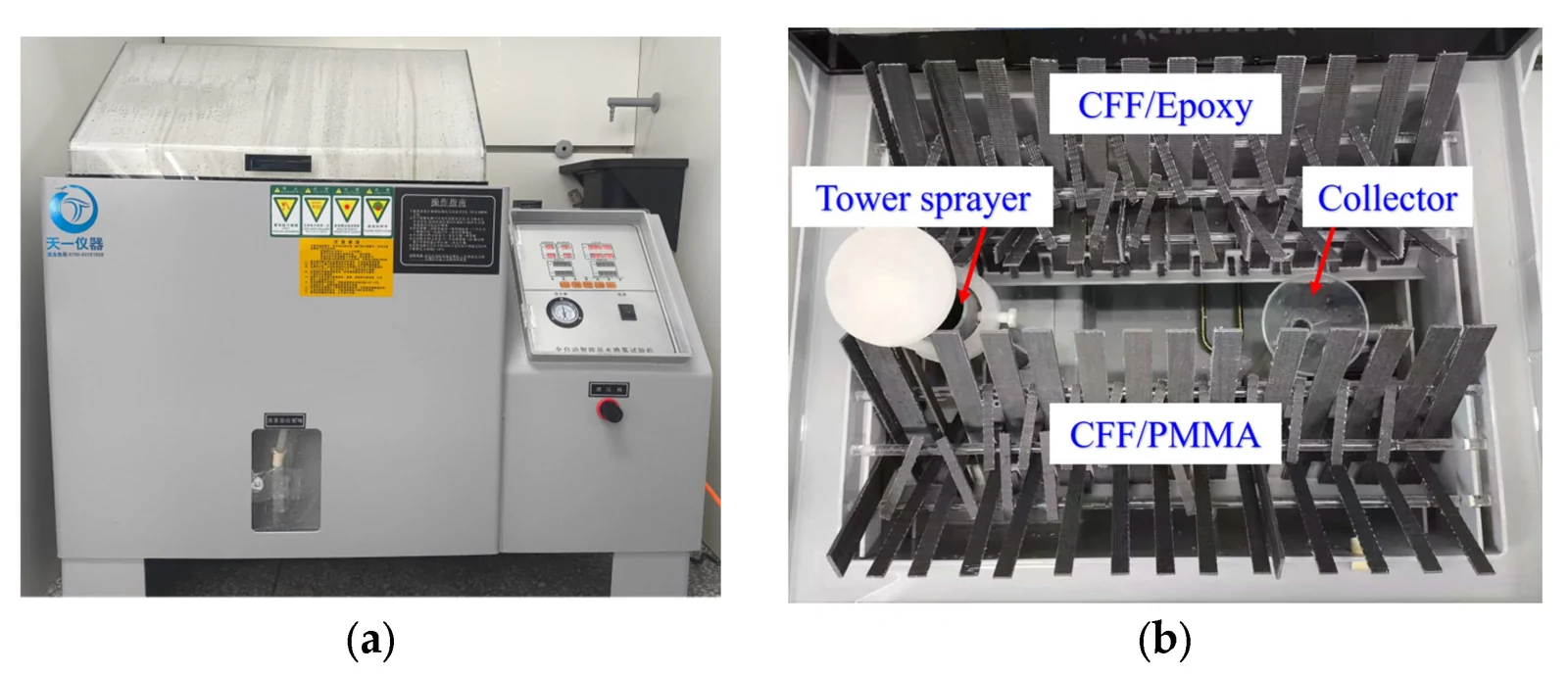
(**a**) The salt spray test chamber and (**b**) internal experimental sample strips.

The salt spray aging test was conducted using a T-YW-60 salt spray test chamber manufactured by Dongguan Tianyi Environmental Testing Instrument Co., Ltd. (Dongguan, China), as shown in Figure 2. The temperature of the pressure barrel was set at 47 ± 1 °C, and the temperatures of both the brine barrel and the salt spray chamber were set at 35 ± 1 °C. The compressed air pressure was maintained at 1.00 ± 0.01 kg/cm^2^ during the test, and the spray volume was controlled at 1.0~2.0 mL/80cm^2^/h to ensure uniform salt spray coverage on the specimen surface.

### 2.4. Performance Testing and Structural Characterization

Specimens were periodically sampled at predefined time intervals during the salt spray aging test. Prior to mechanical tests and characterizations, the samples were dried in a vacuum oven at 60 °C until constant mass was achieved. This procedure removed moisture both on the surface and inside the composites to eliminate the influence of water on the characterization results.

#### 2.4.1. Water Absorption Test

The CF composite specimens were periodically retrieved from the salt spray test chamber according to aging duration, and surface moisture was wiped off with absorbent paper. Subsequently, an electronic analytical balance (OHAUS-AR2140, Shanghai Tianpu Analytical Instrument Co., Ltd. Shanghai, China) with a precision of 0.001 g was employed to measure and record the specimen mass. The water absorption *M_T_* of the specimens was calculated according to Equation (1):

(1) $$M_{T}(\%)=\frac{W_{T}-W_{0}}{W_{0}}\times 100$$

where *W*_0_ is the average mass of five unaged specimens (g) and *W_T_* denotes the average mass of five specimens after aging for time *T* (g).

#### 2.4.2. Mechanical Properties

**Figure 3:**
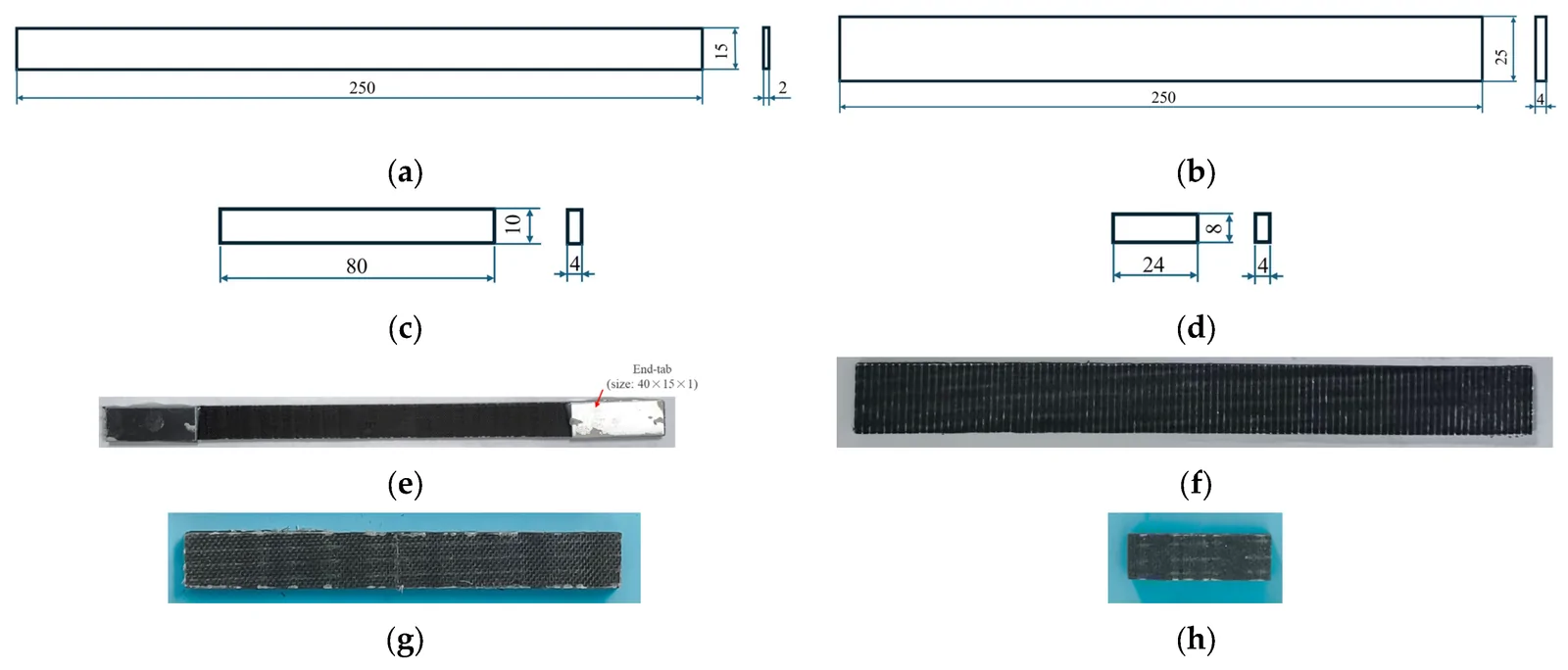
Geometric dimensions and physical photos of test specimens for mechanical characterization of carbon fiber-reinforced composites. (**a**,**e**) Schematic illustration and fabricated sample for 0°tensile testing; (**b**,**f**) schematic representation and prepared specimen for 90°tensile testing; (**c**,**g**) schematic representation and prepared specimen for bending testing; (**d**,**h**) schematic representation and prepared specimen for short-beam shear testing. Dimensions are in mm.

Figure 3 illustrates the geometric configurations of CF composite specimens for tensile, flexural and short-beam shear tests. Mechanical property measurements of CF/PMMA and CF/Epoxy composites were performed on a TENSOR TEST-2 universal testing machine (Model: WDW-50G, Jinan Tianchen Testing Machine Manufacturing Co., Ltd., Jinan, China) equipped with a load capacity of 50 kN. The 0° tensile specimens (Figure 3a,e) were tested at a cross-head speed of 2 mm/min following ISO 527-5 [40], whereas the 90° tensile specimens (Figure 3b,f) were characterized at 2 mm/min in accordance with ISO 527-4 [41]. Furthermore, flexural specimens (Figure 3c,g) and short-beam shear specimens (Figure 3d,h) were tested using a SANSFLEX-MTS mechatronic universal testing machine (Model: CMT 4304, MTS Systems (China) Co., Ltd., Shanghai, China), according to ISO 14125 [42] and ISO 14130 [43], respectively, both at a cross-head speed of 2 mm/min. All experimental data reported in this work represent the average values obtained from five parallel replicate specimens.

#### 2.4.3. Microscopic Characterization

The morphologies of CF composites before and after salt spray aging were observed using a scanning electron microscope (SEM, JSM-5900LV, JEOL Ltd., Tokyo, Japan). The fracture surfaces of flexural and short-beam shear specimens of both composites were also characterized. Specimens were gold-sputtered before observation, and the accelerating voltage was set at 3 kV.

## 3. Water Absorption Behavior and Surface Morphology Evolution

### 3.1. Mechanical Properties (Before Aging)

**Figure 4:**
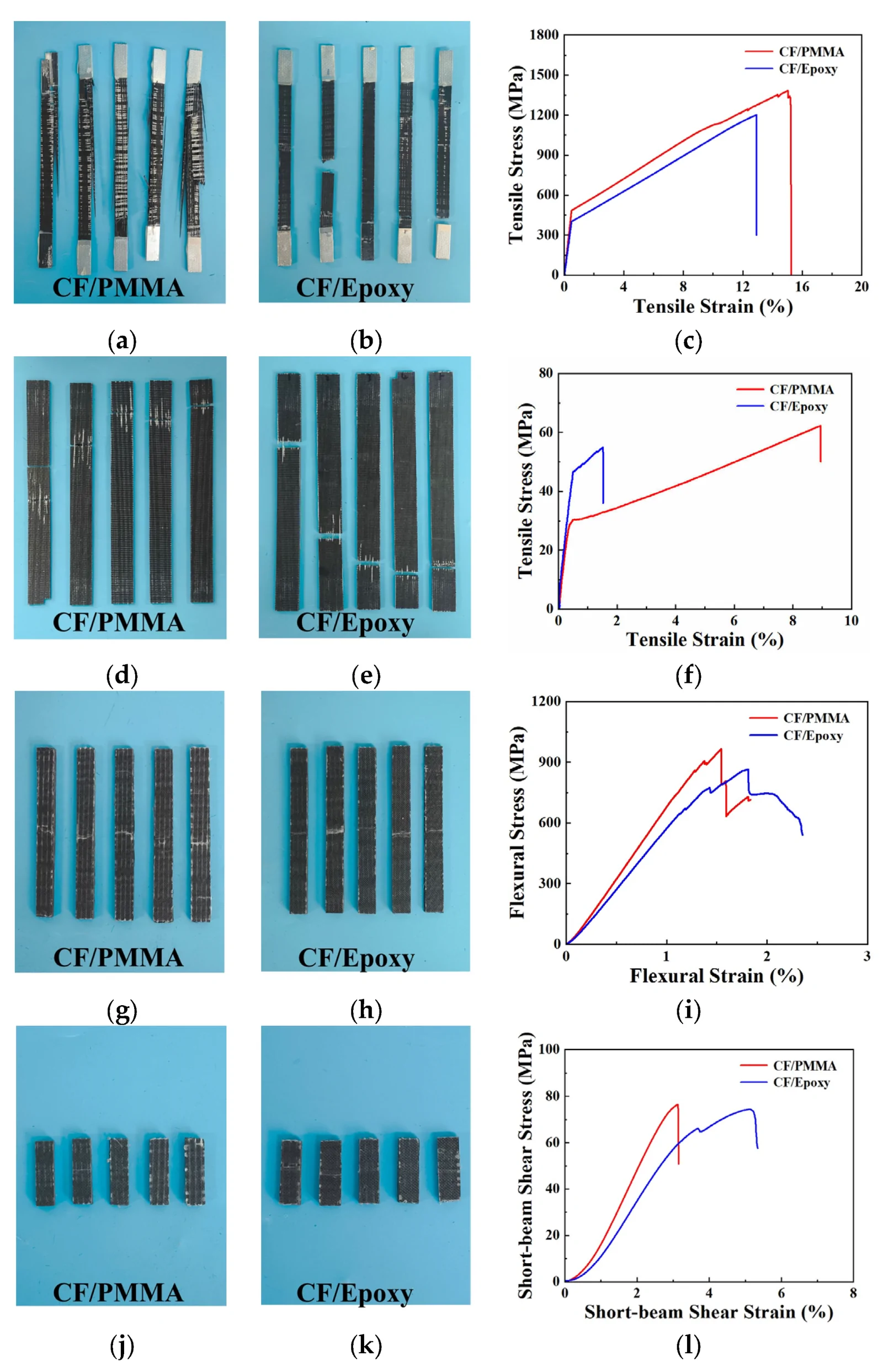
(**a**,**b**) The composites after the 0° tensile test; (**c**) the 0° tensile stress–strain curve; (**d**,**e**) the composites after the 90° tensile test; (**f**) the 90° tensile stress–strain curve; (**g**,**h**) the composites after the bending test; (**i**) the bending stress–strain curve; (**j**,**k**) the composites after the shear test; (**l**) the shear stress–strain curve.

CF/PMMA composites were fabricated via a vacuum-assisted infusion process, while CF/Epoxy composites with an identical architecture and fiber content were prepared as the control group. Prior to salt spray aging, the 0° tensile, 90° tensile, flexural and short-beam shear properties of CF/PMMA and CF/Epoxy were measured to provide baseline references for the subsequent evaluation of aging-induced property evolution. The detailed experimental results are summarized in Table 1, and the failure morphologies together with stress–strain curves of the specimens are displayed in Figure 4.

The test results reveal that for the 0° tensile property along the primary load-bearing fiber direction, CF/PMMA exhibits a tensile strength of 1383.2 MPa, approximately 14.5% higher than that of CF/Epoxy (1208.5 MPa). This indicates a more efficient interfacial load transfer between the PMMA matrix and carbon fiber fabric, which allows the axial load-bearing advantage of carbon fibers to be better exploited. For the 90° tensile property transverse to the fiber direction, which is dominated by matrix and interfacial behaviors, CF/PMMA achieves a tensile strength of 62.2 MPa, about 13.3% higher than that of CF/Epoxy (54.9 MPa). This demonstrates the superior cohesive strength of the PMMA matrix itself, as well as improved fiber–matrix interfacial adhesion.

In terms of flexural and short-beam shear performance, the flexural strength of CF/PMMA reaches 965.1 MPa, around 11.4% higher than that of CF/Epoxy (866.2 MPa). Its shear strength is 76.3 MPa, representing an increment of approximately 3.4% compared with CF/Epoxy (73.8 MPa). Flexural performance reflects the comprehensive mechanical behavior under combined tensile–compressive loading, whereas short-beam shear strength directly characterizes interlaminar interfacial bonding. Collectively, these results suggest that PMMA resin possesses better wettability toward carbon fiber fabrics, leading to tighter interfacial bonding and enhanced resistance against interlaminar delamination and, consequently, superior overall mechanical properties. As can also be observed from the stress–strain curves and failure morphologies in Figure 4, CF/PMMA shows a more stable stress increase and higher load-bearing capacity.

### 3.2. Variation in Water Uptake Behavior

**Figure 5:**
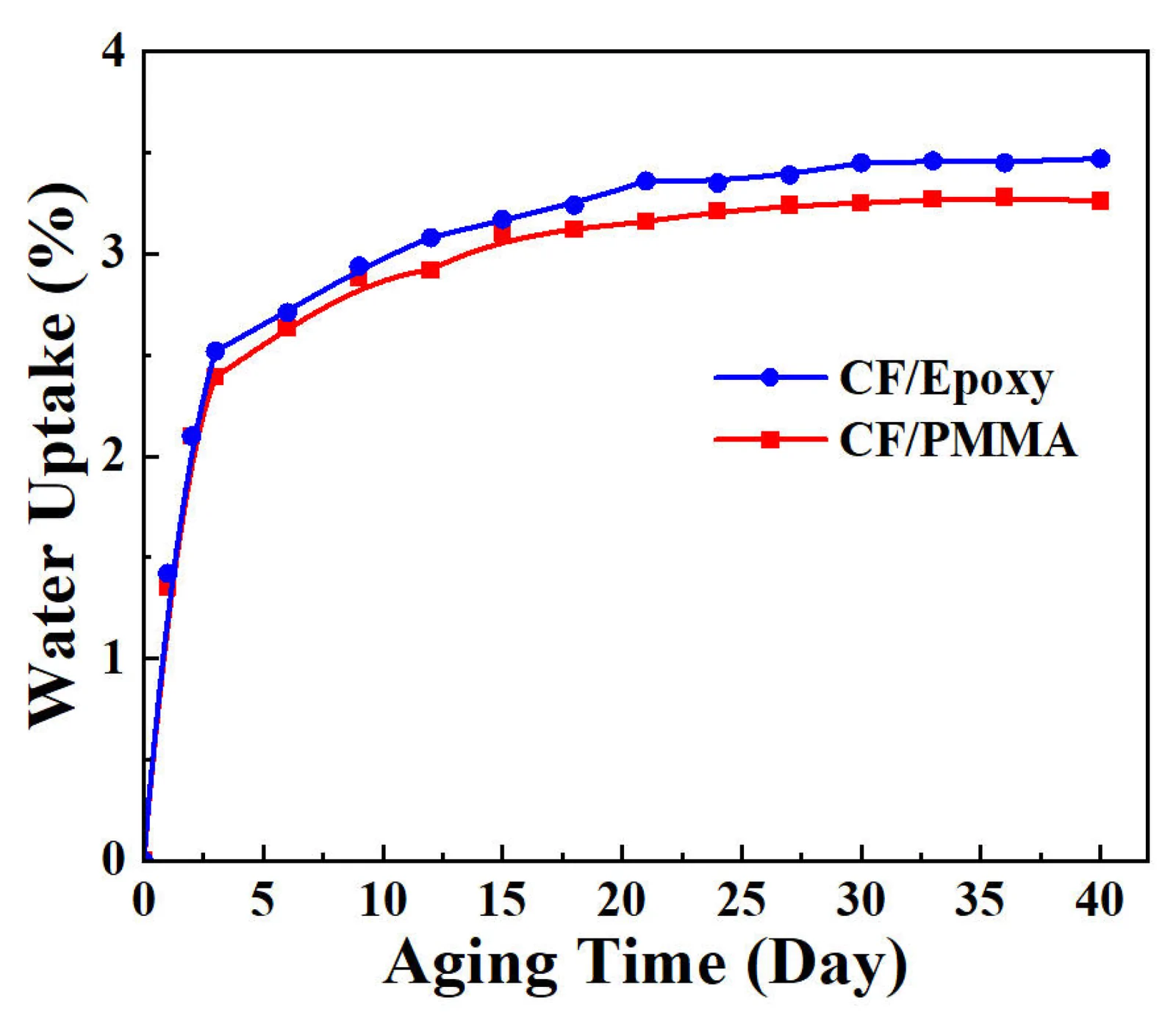
Water uptake of CF composites during salt spray aging.

Figure 5 shows the variation in water uptake with aging time for CF/Epoxy and CF/PMMA composites under salt spray aging conditions. It can be observed that the water uptake evolution of both systems can be divided into two distinct stages. In the first stage, water uptake increases rapidly, presenting an approximately linear rising trend with aging time. In the second stage, the growth rate of water uptake decreases, and the value gradually levels off after 20 days of aging, approaching the moisture-saturation equilibrium state.

During the rapid water uptake stage (Stage I), water molecules rapidly occupy the unfilled inner regions of the CF composites. These unfilled regions consist of free volume within the polymer network, as well as pores located in the matrix and at the fiber–matrix interfaces. Such pores originate from air entrapment, insufficient resin flow, and post-curing reactions during specimen fabrication. An obvious water-concentration gradient forms across the material, driving the diffusion of water molecules into the free volume and pores inside the CF composites. Meanwhile, water molecules can form hydrogen bonds with polar groups on polymer chains, further facilitating water penetration, which accounts for the sharp rise in water uptake in this stage [44,45]. In the second stage, as water gradually saturates the free volume and pores, the water-concentration gradient diminishes and the water uptake rate decreases accordingly. Meanwhile, continuous attack by moisture and corrosive species in the salt spray environment slowly degrades the matrix and interfacial structures, generating additional free volume and pores that provide extra pathways for further water diffusion [46]. Nevertheless, such structural damage is time-dependent and proceeds at a low rate. Consequently, the increment of water uptake is greatly suppressed and finally approaches an equilibrium state.

Comparison of the water uptake behaviors reveals that CF/Epoxy always exhibits higher water uptake than CF/PMMA under identical aging durations, with a distinctly higher equilibrium water uptake. For fiber-reinforced polymer matrix composites, higher water uptake indicates a deeper ingress of water and corrosive agents, which aggravates matrix plasticization, interfacial hydrolysis and interlaminar damage and imposes adverse effects on service performance. Therefore, the lower water uptake directly demonstrates that the CF/PMMA system possesses superior water resistance under salt spray exposure, with less severe water intrusion. The fundamental chemical origin for this discrepancy lies in the abundant strongly polar groups (e.g., hydroxyl and secondary-amine groups) in cured epoxy networks. These groups form strong hydrogen-bonding interactions with water molecules and endow epoxy with stronger water adsorption capacity [47,48,49].

### 3.3. The Surface Morphology After Salt Spray Aging

**Figure 6:**
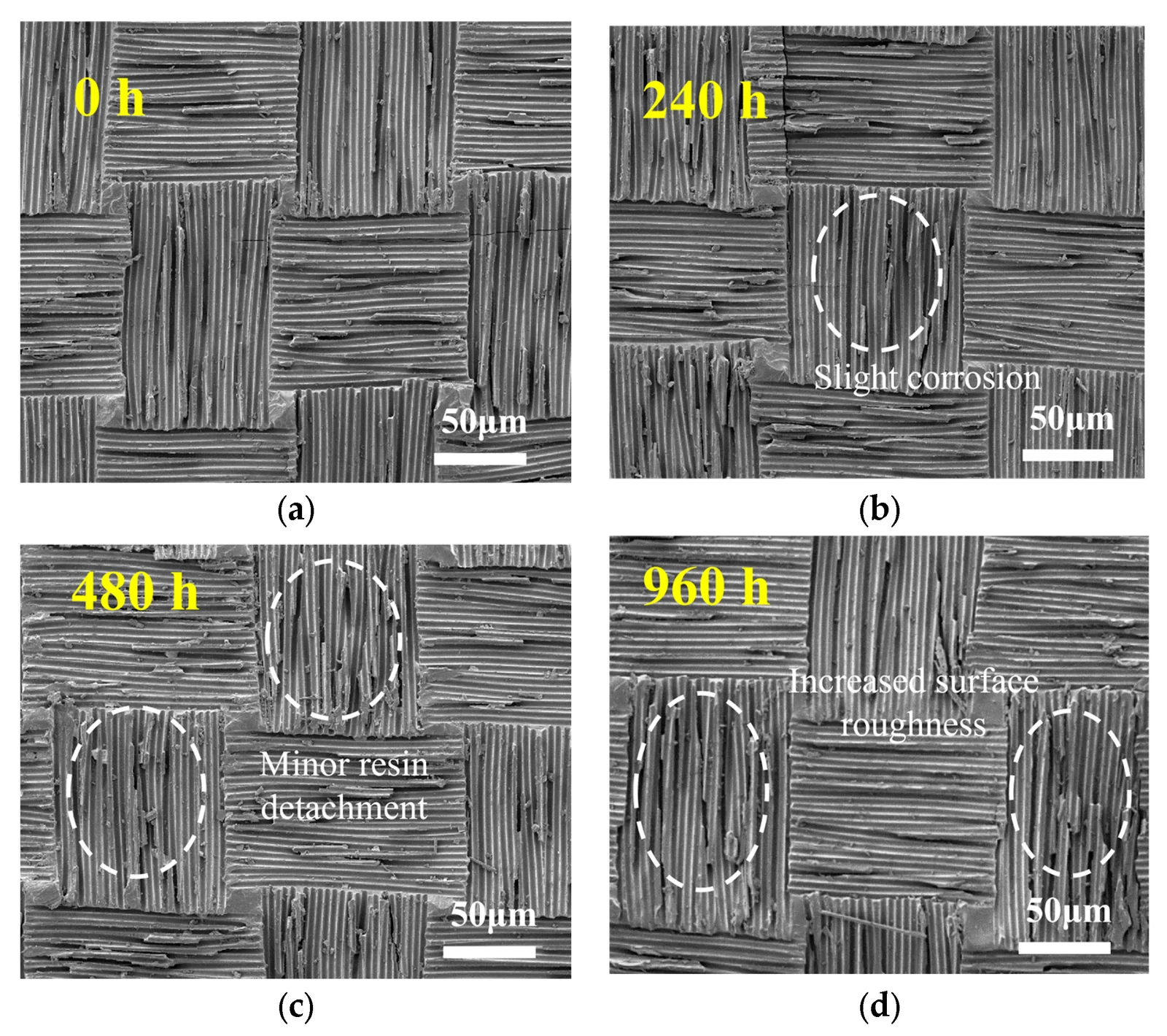
SEM images of CF/PMMA composites under different salt-spray aging durations: (**a**) unaged sample (0 h); (**b**) sample after 240 h aging with slight corrosion; (**c**) sample after 480 h aging with minor resin detachment; (**d**) sample after 960 h aging with increased surface roughness.

**Figure 7:**
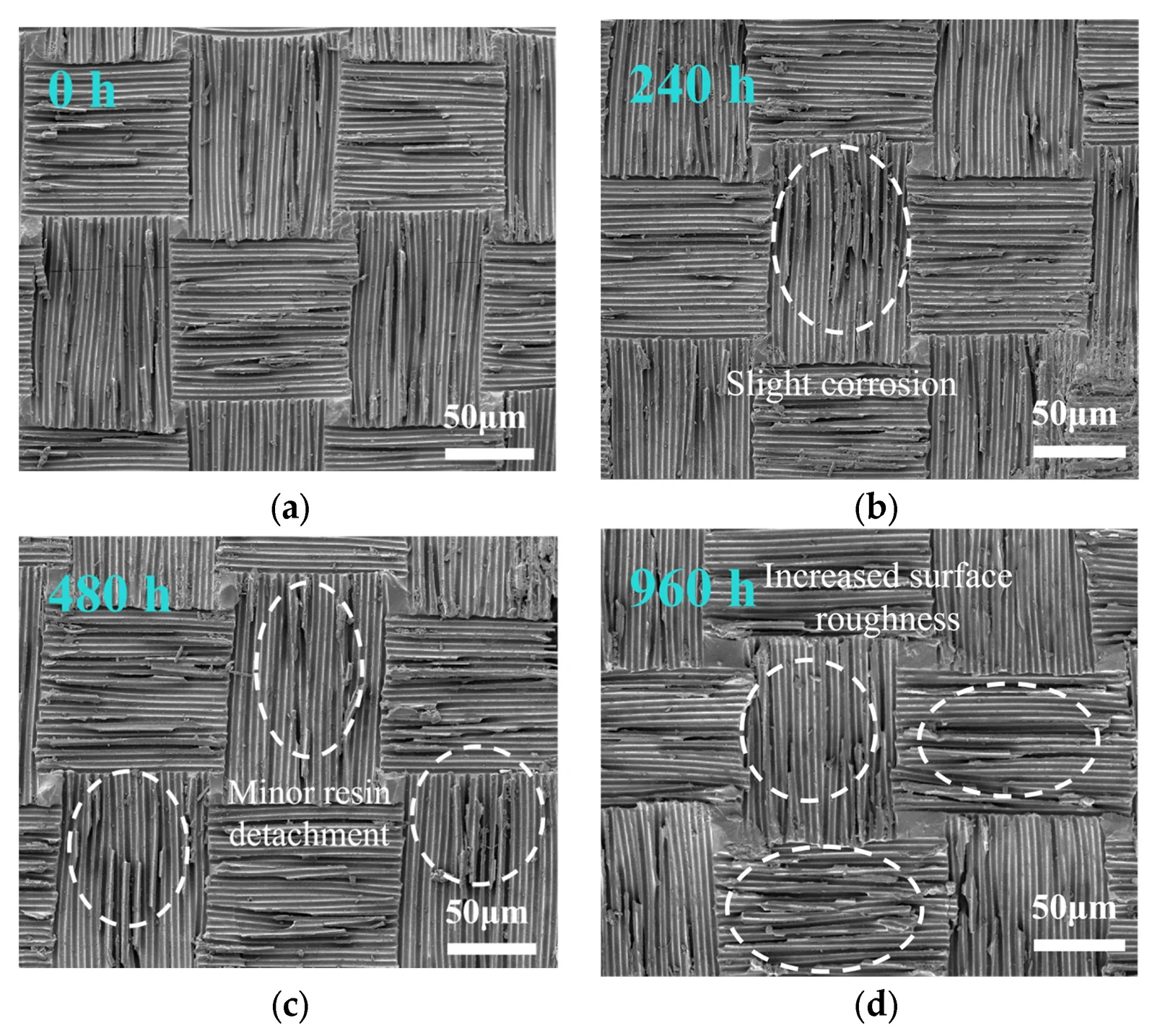
SEM images of CF/Epoxy composites under different salt-spray aging durations: (**a**) unaged sample (0 h); (**b**) sample after 240 h aging with slight corrosion; (**c**) sample after 480 h aging with minor resin detachment; (**d**) sample after 960 h aging with increased surface roughness.

Figure 6 and Figure 7 illustrate the evolution of surface micro-morphologies for CF/PMMA and CF/Epoxy composites after different salt spray aging durations. At 0 h (unaged state), both composites exhibit well-ordered microstructures. Carbon fiber tows are fully wetted and encapsulated by the resin matrix, and resin uniformly fills the gaps between fiber tows. The surfaces are relatively smooth and dense, without obvious exposed fibers or macroscopic crack defects.

As salt spray aging extends to 240 h, the resin matrix on CF/PMMA and CF/Epoxy still maintains good continuity, and the woven architecture remains intact and distinct. Only slight erosion occurs for the resin at the edges of fiber tows; no obvious corrosion pits or exposed fibers are observed.

At the aging duration of 480 h, the surface roughness of CF/PMMA and CF/Epoxy becomes visible, and surface erosion marks grow more numerous. Nevertheless, the resin layer still retains its overall continuity without large-area resin spalling, and the general morphology of the woven structure remains intact. For CF/Epoxy, surface microcracks propagate along fiber-tow directions and interconnect with one another. Substantial resin loss occurs between fiber tows, and, compared with CF/PMMA, the degree of tow exposure for the carbon fiber increases remarkably.

Upon completion of 960 h aging, the difference in surface damage between the two materials is further enlarged. Obvious erosion and roughening appear on the CF/PMMA surface. Resin wears off at the top of partial fiber tows, and a small number of fibers are exposed. However, fiber tows are still partially covered by resin, no large-scale interfacial debonding occurs, and the overall framework of the woven structure is preserved. In comparison, CF/Epoxy suffers extensive surface resin loss, with heavily exposed carbon fiber tows. The gaps between fiber tows are significantly widened, severe interfacial debonding can be observed, and the surface-layer resin matrix is severely damaged.

Overall, CF/PMMA undergoes less severe surface corrosion than CF/Epoxy during salt spray aging, demonstrating superior surface salt spray resistance and interfacial stability.

## 4. Effects of Salt Spray Aging on the Performance of CF/PMMA

Mechanical performance serves as a critical indicator for evaluating the aging resistance of composite materials. In this section, the mechanical property evolution of the two composites under different salt spray aging durations is investigated in terms of 0° tensile, 90° tensile, flexural and short-beam shear performance. The detailed numerical data are summarized in Table 2 and Table 3.

### 4.1. 0° Tensile Test

**Figure 8:**
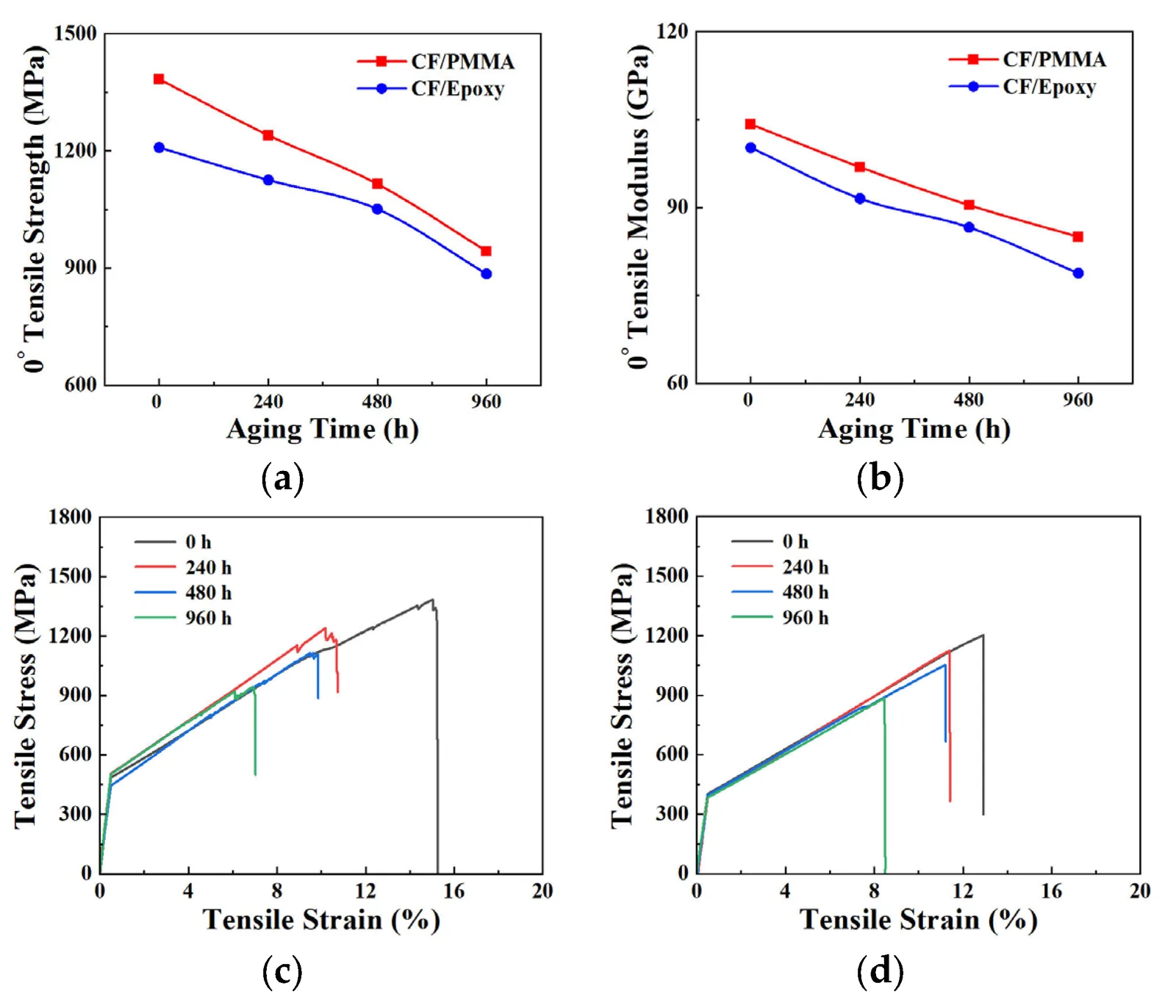
(**a**) 0° tensile strength of the two composites; (**b**) 0° tensile modulus of the two composites; (**c**) stress–strain curves of CF/PMMA; (**d**) stress–strain curves of CF/Epoxy.

In compliance with the ISO 527-5 standard, 0° tensile tests were conducted on the salt spray-aged CF/PMMA and CF/Epoxy composites, and the results are summarized in Figure 8. Both the 0° tensile strength and tensile modulus of the two composites exhibit a continuous declining trend with increasing salt spray aging duration. For the CF/PMMA composite, the initial 0° tensile strength is 1383.2 MPa. After 960 h of salt spray aging, its strength drops to 943.2 MPa, corresponding to a strength reduction of 31.81% and a strength retention rate of 68.19%. The tensile modulus decreases from the initial value of 104.2 GPa to 85.0 GPa, with a modulus retention rate of 81.57%. As for the CF/Epoxy composite, its 0° tensile strength decreases from 1208.5 MPa to 885.3 MPa, showing a strength loss of 26.74% and a strength retention rate of 73.26%. Its tensile modulus falls from 100.2 GPa to 78.8 GPa, yielding a modulus retention rate of 78.64%.

In terms of the magnitude of property degradation, CF/PMMA presents a faster strength reduction rate than CF/Epoxy in the early aging stage, whereas its degradation rate slows down in the later aging period (480–960 h). By contrast, CF/Epoxy displays an approximately linear strength decay throughout the whole aging process. Because 0° tensile performance is predominantly governed by carbon fibers, salt spray aging imposes a relatively limited influence on the intrinsic properties of carbon fibers [32]. The strength degradation mainly originates from the weakened support and load transfer capability of the matrix toward fibers [5].

Compared with the published literature data, Li et al. [33] reported that the maximum reduction in 0° tensile strength of CFRP laminates was approximately 11.5% after 7-month seawater immersion at 30 °C. The more remarkable degradation of 0° tensile strength after 960 h of accelerated salt spray aging in the present study can be mainly attributed to the continuous high humidity and high-chloride-ion environment in artificial accelerated salt spray tests, which aggravates the interfacial degradation of composites.

### 4.2. 90° Tensile Test

**Figure 9:**
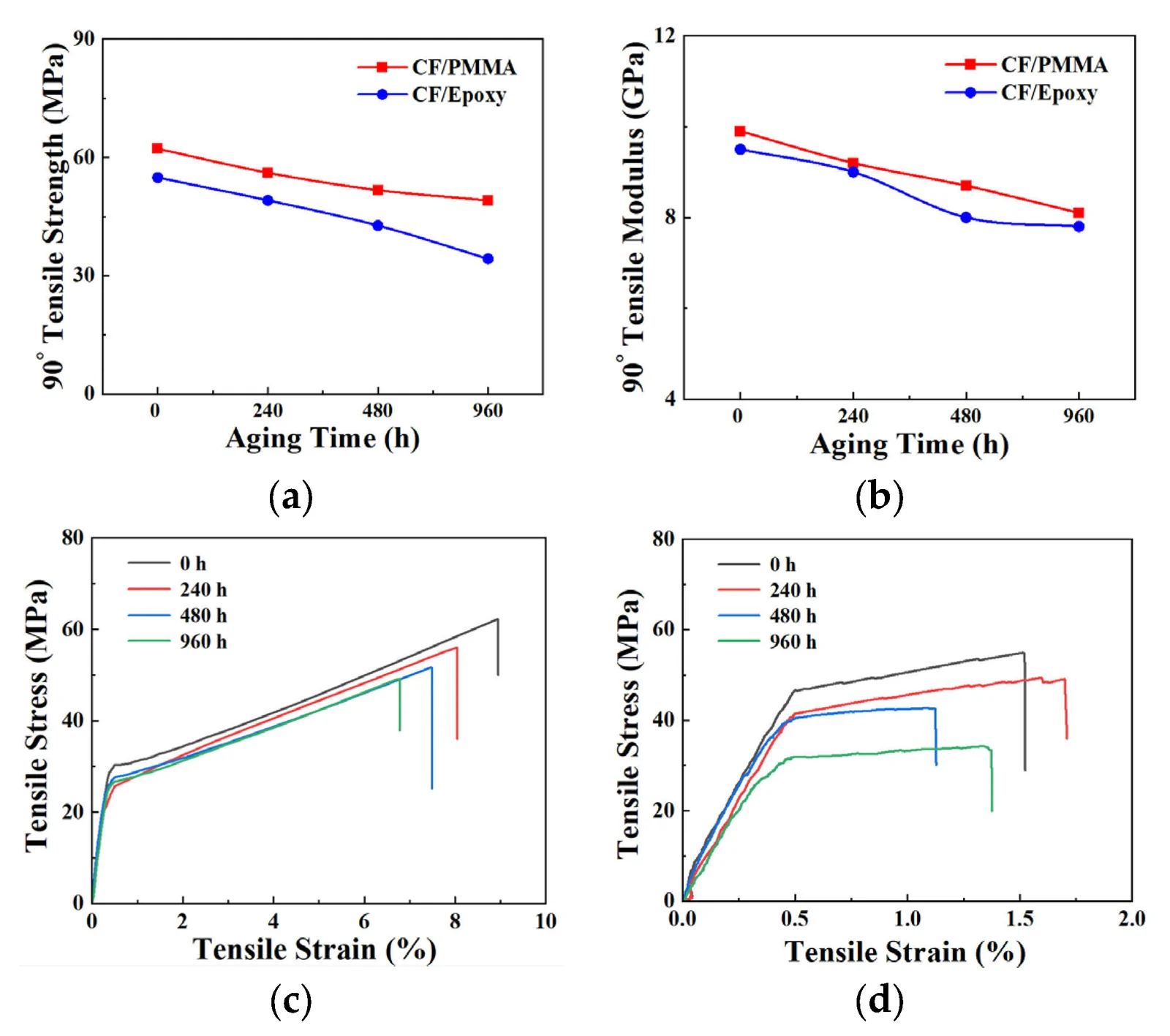
(**a**) 90° tensile strength of the two composites; (**b**) 90° tensile modulus of the two composites; (**c**) stress–strain curves of CF/PMMA; (**d**) stress–strain curves of CF/Epoxy.

In accordance with ISO 527-4, 90° tensile tests were performed, and the corresponding results are presented in Figure 9. The test results show that both the 90° tensile strength and modulus of the two materials continuously decrease with increasing aging duration. For the CF/PMMA composite, the initial 90° tensile strength is 62.2 MPa. After 960 h of salt spray aging, the strength drops to 49.1 MPa, corresponding to a strength reduction of 21.06% and a strength retention rate of 78.94%. Its tensile modulus decreases from the initial value of 9.9 GPa to 8.1 GPa, with a modulus retention rate of 81.82%. For the CF/Epoxy composite, the 90° tensile strength decreases from 54.9 MPa to 34.6 MPa, exhibiting a strength loss of 36.98% and a low strength retention rate of only 63.02%. Its tensile modulus falls from 9.5 GPa to 7.8 GPa, giving a modulus retention rate of 82.11%.

The comparison reveals that the degradation magnitude of 90° tensile strength for CF/Epoxy after 960 h aging (36.98%) is remarkably larger than that of CF/PMMA (21.06%), which demonstrates the superior salt spray corrosion stability of the PMMA matrix. Strong interfacial bonding can be formed by chemical bonds between epoxy resin and polar groups (e.g., carboxyl and hydroxyl groups) on carbon fiber surfaces; nevertheless, such polar interfaces are susceptible to hydrolytic fracture under the effect of water molecules [6]. By contrast, the interfacial adhesion between PMMA and carbon fibers mainly relies on physical adsorption and weak van der Waals forces, so water molecules impose relatively limited damage to this physical-dominated interface [22]. Furthermore, the lower water uptake of PMMA also contributes to maintaining interfacial integrity.

### 4.3. Flexural Test

**Figure 10:**
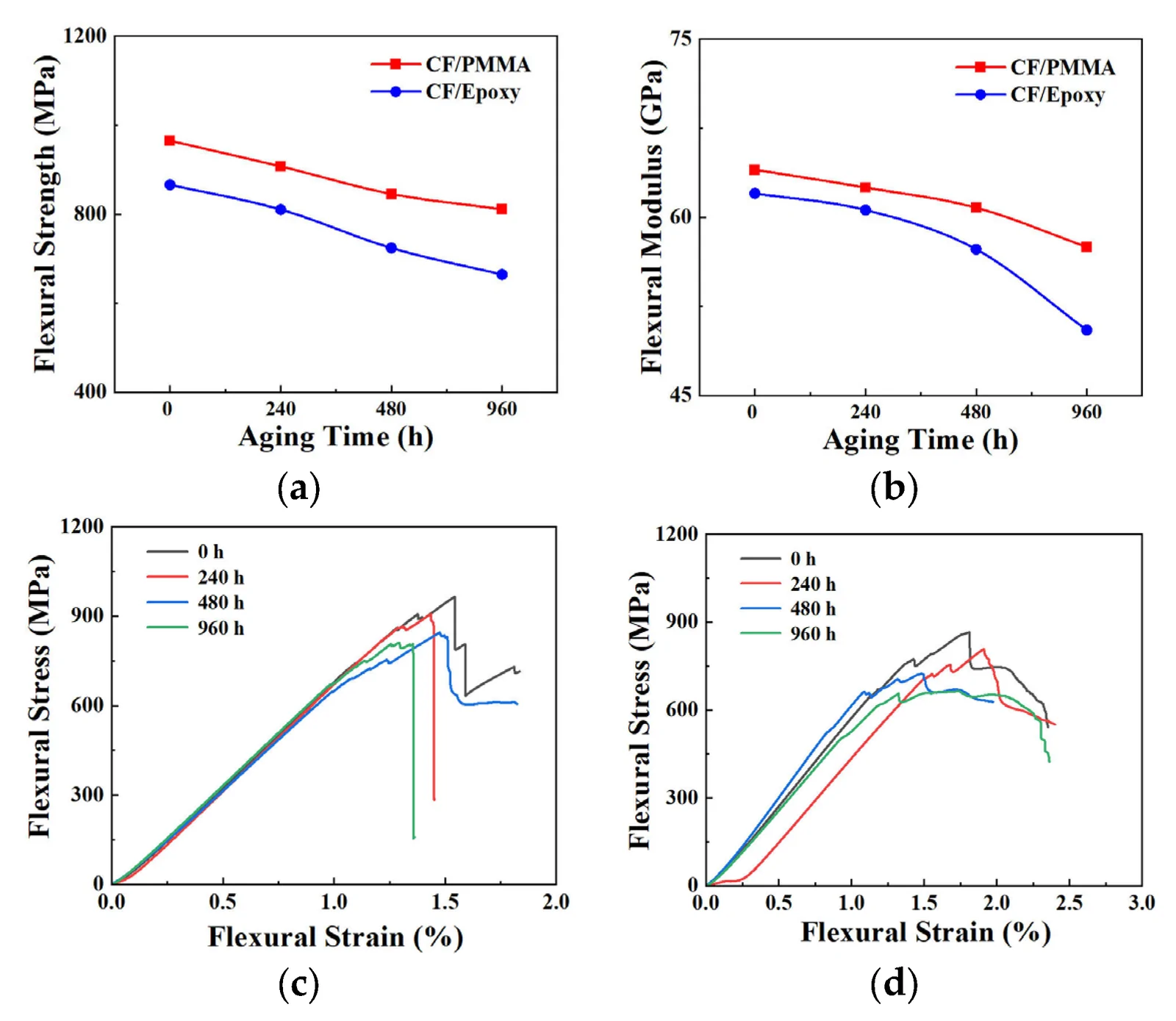
(**a**) Flexural strength of the two composites; (**b**) flexural modulus of the two composites; (**c**) flexural stress–strain curves of CF/PMMA; (**d**) flexural stress–strain curves of CF/Epoxy.

Three-point flexural tests were performed according to ISO 14125 [42], and the results are presented in Figure 10. The test results demonstrate that the initial flexural strength of the CF/PMMA composite is 965.1 MPa. After 960 h of salt spray aging, its strength decreases to 811.0 MPa, corresponding to a strength reduction of 15.97% and a flexural strength retention rate of 84.03%. The flexural modulus drops from the initial value of 64.0 GPa to 57.5 GPa, with a modulus loss of 10.16%. For the CF/Epoxy composite, the flexural strength decreases from 866.2 MPa to 664.3 MPa, showing a strength degradation of 23.31% and a strength retention rate of 76.69%. Its flexural modulus falls from 62.0 GPa to 50.5 GPa, with a modulus reduction of 18.55%.

Compared with the available research, Yan et al. [50] reported that composites exhibited flexural strength reductions ranging from 9.3% to 23.5% and flexural modulus reductions of 13.9–25.2% after one-year immersion in seawater, natural water and alkaline solutions. The degradation magnitude of the flexural properties of CF/PMMA in this work lies at the lower bound of this range, which further confirms the superior property retention capability of PMMA matrix composites under salt spray conditions.

**Figure 11:**
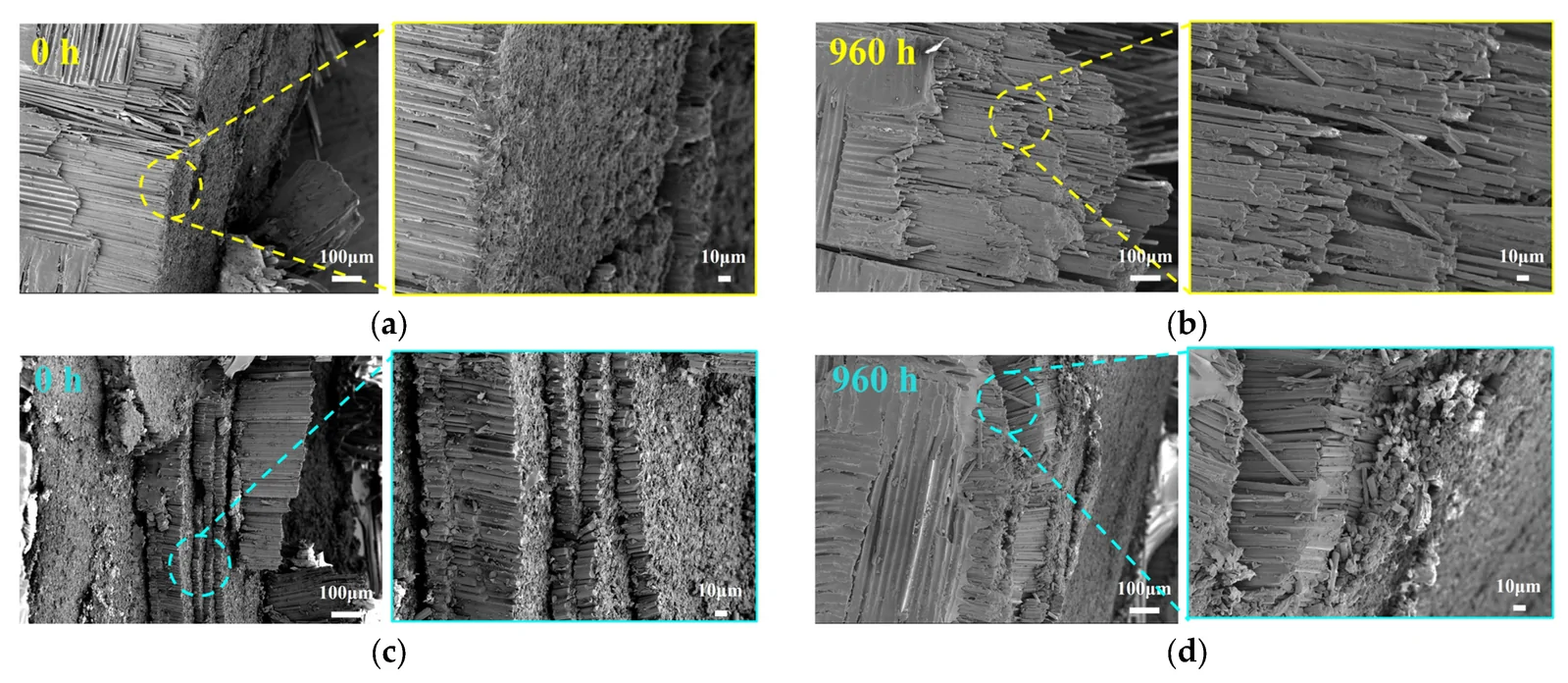
SEM micrographs of fracture regions of the two composites after flexural tests. (**a**) Fracture surface and local magnified view of unaged CF/PMMA composite; (**b**) fracture surface and local magnified view of CF/PMMA composite after 960 h salt spray aging; (**c**) fracture surface and local magnified view of unaged CF/Epoxy composite; (**d**) fracture surface and local magnified view of CF/Epoxy composite after 960 h salt spray aging.

Figure 11 presents the SEM micrographs of the fracture surfaces for the two composites after the flexural tests. The fracture surface of the unaged CF/PMMA specimen (Figure 11a) exhibits typical ductile fracture characteristics. The resin matrix fully encases the fibers with short fiber pull-out lengths, and abundant resin adheres to fiber surfaces on the fracture section, indicative of favorable interfacial bonding. After 960 h of salt spray aging (Figure 11b), fiber–matrix interfacial debonding and fiber exposure are observed on the CF/PMMA fracture surface, and microcracks emerge within the local matrix regions. Nevertheless, a certain level of interfacial-bonding strength is still maintained overall.

In comparison, the fracture surface of unaged CF/Epoxy (Figure 11c) also shows satisfactory fiber–matrix adhesion. However, after 960 h aging (Figure 11d), extensive interfacial debonding, remarkably increased fiber pull-out length and a fragmented resin matrix can be found on the fracture surface, which indicates severe interfacial deterioration.

### 4.4. Short-Beam Shear Test

**Figure 12:**
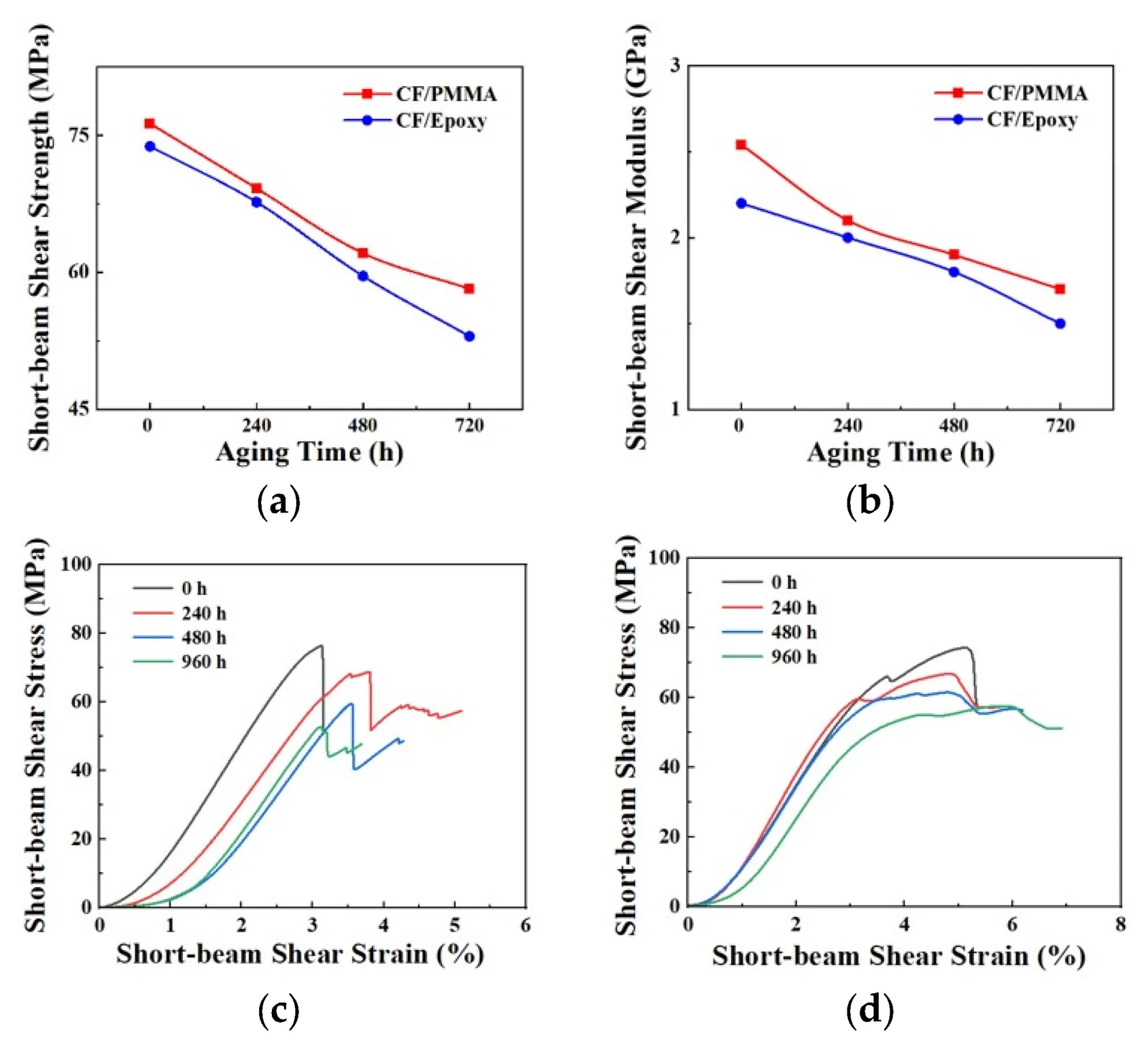
(**a**) Short-beam shear strength of the two composites; (**b**) short-beam shear modulus of the two composites; (**c**) short-beam shear stress–strain curves of CF/PMMA; (**d**) short-beam shear stress–strain curves of CF/Epoxy.

Short-beam shear tests were carried out following ISO 14130, and the corresponding results are presented in Figure 12. In the unaged state, the shear strength of the CF/PMMA composite reaches 76.3 MPa, which is 3.39% higher than that of CF/Epoxy (73.8 MPa). After 960 h of salt spray aging, the shear strength of CF/PMMA decreases to 58.2 MPa, representing a strength reduction of 23.72%. By comparison, the shear strength of CF/Epoxy drops to 53.0 MPa, with a strength loss of 28.18%. Comparative analysis reveals that CF/PMMA exhibits a higher short-beam shear strength retention rate (76.28%) than CF/Epoxy (71.82%).

Li et al. [7] reported that carbon fiber-reinforced epoxy composites achieved short-beam shear strength retention rates of 73.75% for NOL-ring specimens and 84.10% for laminate specimens after 90-day aging under high-temperature and high-concentration salt spray conditions. The shear strength retention rate of CF/PMMA in the present study falls within this reference range, whereas CF/Epoxy delivers a slightly lower retention rate. This may be attributed to the high susceptibility of epoxy matrix to moisture-induced hydrolysis under salt spray exposure.

**Figure 13:**
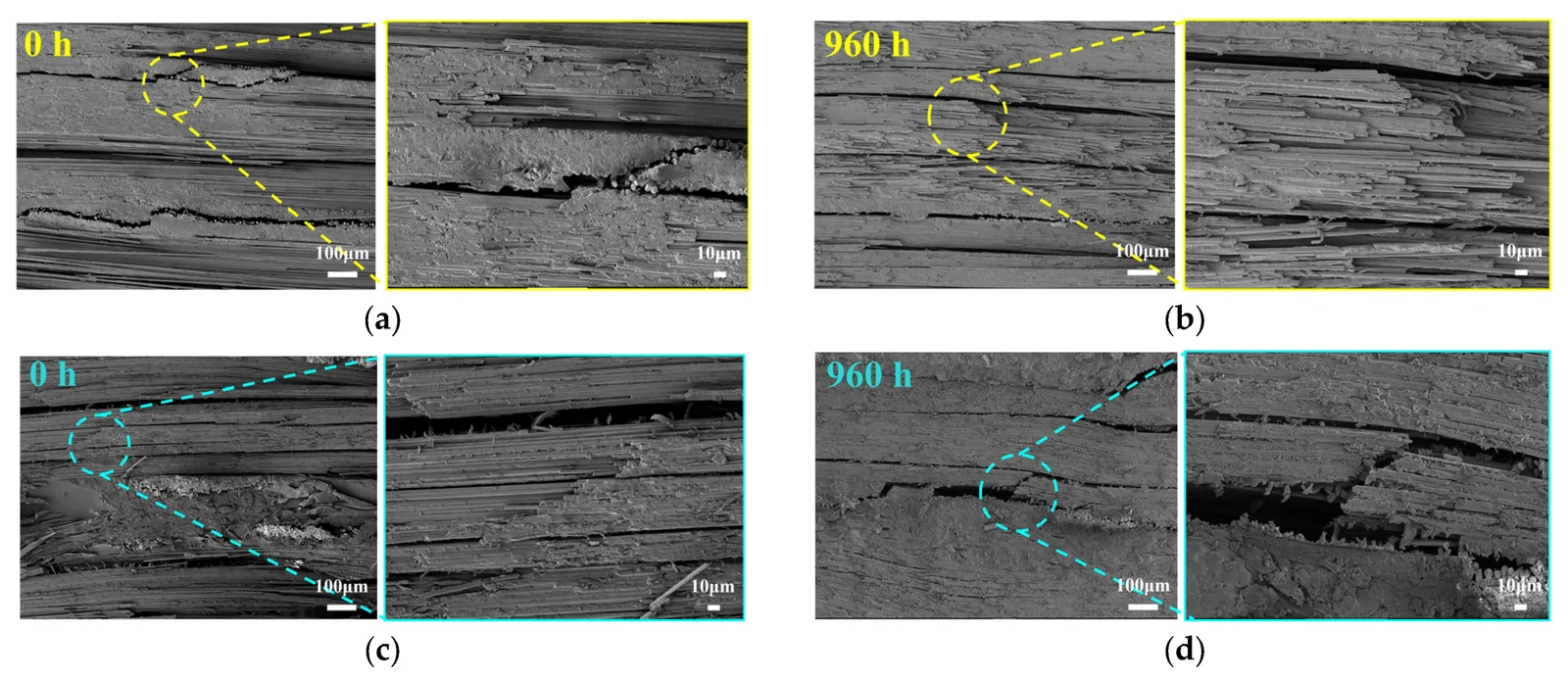
SEM micrographs of fracture regions of the two composites after short-beam shear tests. (**a**) Fracture surface and local magnified view of unaged CF/PMMA composite; (**b**) fracture surface and local magnified view of CF/PMMA composite after 960 h salt spray aging; (**c**) fracture surface and local magnified view of unaged CF/Epoxy composite; (**d**) fracture surface and local magnified view of CF/Epoxy composite after 960 h salt spray aging.

Figure 13 shows the SEM micrographs of the fracture surfaces for the two composites after the short-beam shear tests. For the unaged CF/PMMA (Figure 13a), the fibers are neatly arranged, and the matrix fills the interior densely. The fracture surface displays typical short-beam shear failure features, and residual resin layers on the fiber surfaces indicate high interfacial-bonding strength. After 960 h of salt spray aging (Figure 13b), obvious fiber–matrix debonding and interlaminar cracking are observed on the CF/PMMA fracture surface; nevertheless, partial resin residues still remain on the fiber surfaces.

The unaged CF/Epoxy (Figure 13c) exhibits favorable interfacial adhesion. By contrast, after 960 h of aging (Figure 13d), the fracture surface suffers severe interlaminar delamination and fully exposed fibers, which demonstrates the remarkable interfacial degradation of the epoxy matrix induced by salt spray aging.

## 5. Lifetime Prediction

The aging behavior of composite materials under salt spray environments is governed by multiple influencing factors. Long-term natural exposure aging tests for composites require extremely lengthy experimental periods [51,52]. For this reason, regression analysis is widely used to study the time evolution of mechanical properties. In this study, the median strength aging equation proposed by Gunyaev et al. [53] is used to predict the lifetime of the CF composites. The mathematical expression of the model is given as follows:

(2) $$S_{f}=S_{0}+\eta (1-e^{-\lambda t})-\beta \ln(1+\theta t)$$

where *S_f_* is the mechanical strength value at aging time *t*; *S*_0_ is the initial mechanical strength; and *η*, *λ*, *β* and *θ* are undetermined parameters.

Then, with the method of pluralistic dichotomy [51], the parameter values of *S*_0_, *η*, *β*, *λ* and *θ* for the tensile and shear strength data in Table 2 and Table 3 were obtained.

**Figure 14:**
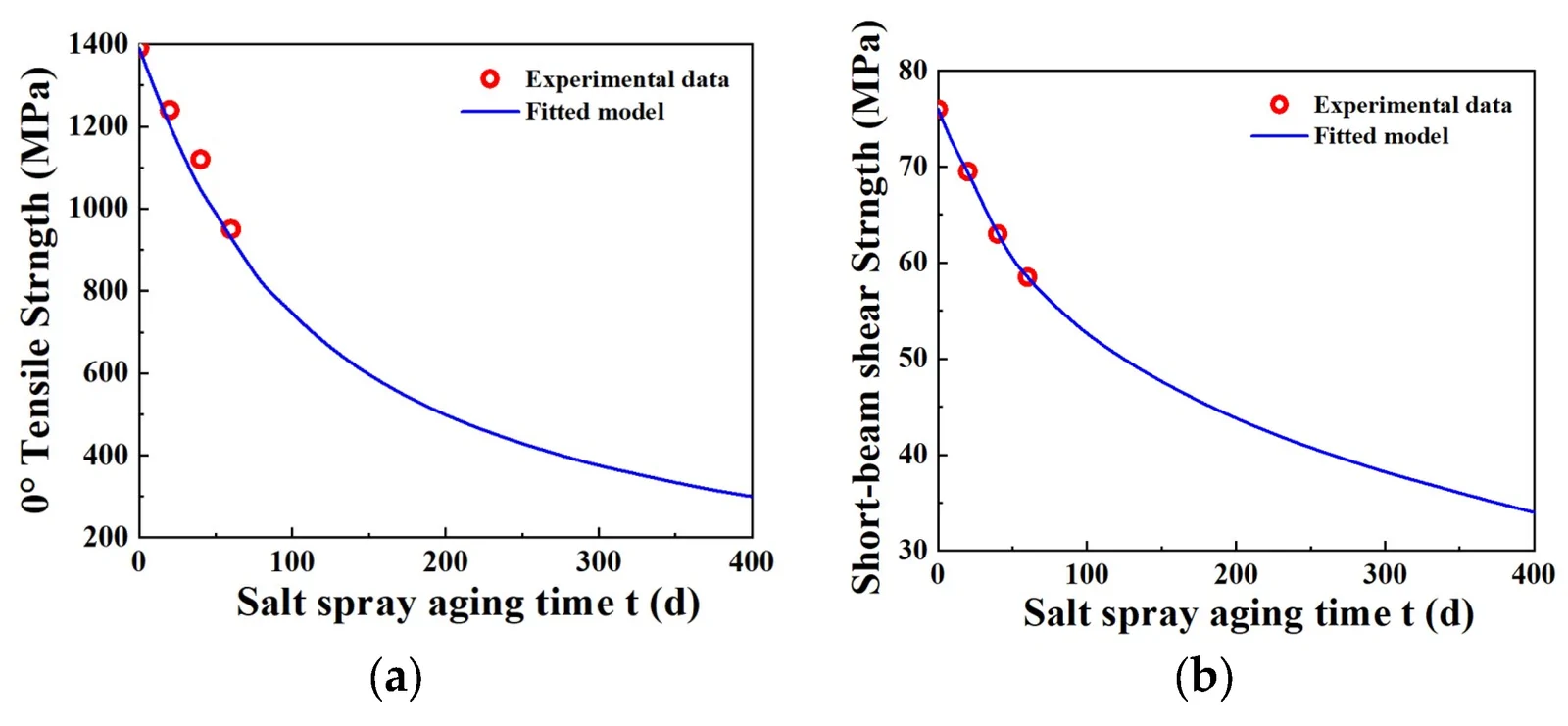
Fitted curves of tensile and shear strength for CF/PMMA composites: (**a**) tensile residual strength curve with aging time; (**b**) shear residual strength curve with aging time.

As presented in Figure 14, the median curves exhibited a significant fit at the aging time of 0 to 40 days. The fitting equations are shown in Equations (3) and (4), and the correlation coefficients R^2^ were 98.40% and 97.60%, respectively.

(3) $$S_{t}=1383.2+232.03(1-e^{-7.91\times 10^{-3}t})-298.81\ln(1+8.81\times 10^{-3}t)$$

(4) $$S_{s}=76.3+49.99(1-e^{-2.96\times 10^{-4}t})-28.39\ln(1+2.01\times 10^{-3}t)$$

To date, there are still no reliable models or empirical formulas to describe the equivalence relationship between accelerated aging and natural environmental aging for life prediction purposes regarding CFRP and GFRP in natural environments. Accordingly, the service life prediction of CF/PMMA composites proposed in this paper is established exclusively based on the experimental conditions presented in the preceding sections of this work. As summarized in Table 4, the predicted service lives corresponding to 80% residual tensile strength and residual shear strength reach 499.45 days and 659.42 days, respectively.

It can be observed that the predicted service life under shear loading is longer than that under tensile loading at the same strength retention threshold. Such a discrepancy mainly originates from the different damage evolution mechanisms under tensile and shear states. Tensile loading is more sensitive to matrix cracking and fiber–matrix interface debonding induced by hygrothermal aging, which accelerates the degradation of tensile performance. By contrast, shear performance is dominated by matrix properties and interfacial bonding, showing relatively slower degradation rates under identical aging environments.

If the threshold of 50% strength retention is defined as the material failure criterion as proposed in Ref. [51], the estimated service lifetimes of CF/PMMA composites under tension and shear modes amount to 99.05 days and 389.06 days, respectively. Compared with the 80% strength retention condition, the service life is sharply reduced when the 50% failure threshold is adopted, which demonstrates that the allowable strength retention criterion exerts a remarkable influence on the final lifetime prediction results.

It should be emphasized that the above-mentioned prediction results were obtained under the given accelerated aging conditions. Because the conversion rule between accelerated aging and real-service natural environmental aging has not been well established, these predicted values can only serve as a reference for material durability evaluation rather than absolute service life indexes for practical engineering applications. Further investigations are required to construct the correlation between accelerated aging test data and long-term natural exposure results to ultimately realize more accurate lifetime assessment for CF/PMMA composites in real-world service scenarios.

## 6. Conclusions

Static mechanical characterization reveals that CF/PMMA laminates outperform CF/Epoxy in the tested mechanical indexes. The 0° tensile, 90° tensile, flexural and short-beam shear strengths of CF/PMMA are higher by 14.46%, 13.30%, 11.42% and 3.39%, respectively. The water absorption by both composites presents a two-stage evolution law under salt spray exposure: a rapid linear rising phase within the first 20 days, followed by a slow saturation stage.

Salt spray aging continuously degrades all mechanical properties of the two composite systems. After 960 h of salt spray aging, the strength reduction rates of CF/PMMA in terms of 0° tensile, 90° tensile, flexural and shear strengths are 31.81%, 21.06%, 15.97% and 23.72%, respectively. The corresponding values for CF/Epoxy are 26.74%, 36.98%, 23.31% and 28.18%, respectively, yet CF/PMMA maintains a significantly higher residual strength retention after 960 h of exposure. SEM surface and fracture morphology analysis identifies the core salt spray degradation mechanism: moisture and chloride ions erode the matrix and trigger progressive fiber–matrix debonding and interlayer cracking. CF/PMMA retains continuous matrix coverage on carbon fibers and limited interlayer separation after long-term aging, whereas CF/Epoxy suffers massive resin spalling, severe fiber exposure and extensive interlaminar delamination.

Based on the lifetime prediction curves of the CF/PMMA, the service lives corresponding to the 50% residual strength threshold are predicted as 99.05 days (0° tension) and 389.06 days (short-beam shear), offering quantitative life evaluation support for marine structural design.

This work systematically compares the salt spray durability of recyclable thermoplastic CF/PMMA and conventional thermoset CF/Epoxy composites, verifies the superior anti-corrosion potential of PMMA matrix carbon fabric composites in salt-rich marine environments, and establishes a residual strength prediction model for thermoplastic composite components under accelerated salt spray aging conditions.

## Figures and Tables

**Table 1:** Mechanical properties of CF/PMMA and CF/Epoxy.

Performance	CF/PMMA	CF/Epoxy
0° Tensile Strength (MPa)	1383.2 ± 15.2	1208.5 ± 18.1
0° Tensile Modulus (GPa)	104.2 ± 3.4	100.2 ± 2.7
90° Tensile Strength (MPa)	62.2 ± 1.4	54.9 ± 1.5
90° Tensile Modulus (GPa)	9.9 ± 0.4	9.5 ± 0.3
Flexural Strength (MPa)	965.1 ± 14.1	866.2 ± 12.7
Flexural Modulus (GPa)	64.0 ± 2.4	62.0 ± 1.7
Short-beam Shear Strength (MPa)	76.3 ± 2.0	73.8 ± 1.8
Short-beam Shear Modulus (GPa)	2.5 ± 0.05	2.2 ± 0.06

**Table 2:** Mechanical properties of CF/PMMA composites after salt spray aging.

Mechanical Properties	Unit	Salt Spray Test Duration			
		0 h	240 h	480 h	960 h
0° Tensile Strength	(MPa)	1383.2 ± 15.2	1240.1 ± 12.8	1115.1 ± 17.3	943.2 ± 10.6
0° Tensile Modulus	(GPa)	104.2 ± 3.4	96.9 ± 3.6	90.4 ± 3.8	85.0 ± 3.6
90° Tensile Strength	(MPa)	62.2 ± 1.4	56.1 ± 1.6	51.7 ± 2.0	49.1 ± 1.8
90° Tensile Modulus	(GPa)	9.9 ± 0.4	9.2 ± 0.5	8.7 ± 0.5	8.1 ± 0.4
Flexural Strength	(MPa)	965.1 ± 14.1	907.4 ± 9.4	845.5 ± 13.6	811.0 ± 11.8
Flexural Modulus	(GPa)	64.0 ± 2.4	62.5 ± 2.6	60.8 ± 2.5	57.5 ± 2.3
Short-beam Shear Strength	(MPa)	76.3 ± 2.0	69.2 ± 1.8	62.8 ± 1.9	58.2 ± 1.6
Short-beam Shear Modulus	(GPa)	2.5 ± 0.05	2.1 ± 0.03	1.9 ± 0.06	1.7 ± 0.06

**Table 3:** Mechanical properties of CF/Epoxy composites after salt spray aging.

Mechanical Properties	Unit	Salt Spray Test Duration			
		0 h	240 h	480 h	960 h
0° Tensile Strength	(MPa)	1208.5 ± 18.1	1125.3 ± 13.4	1051.1 ± 15.9	885.3 ± 14.5
0° Tensile Modulus	(GPa)	100.2 ± 2.7	91.5 ± 3.0	86.6 ± 3.2	78.8 ± 3.4
90° Tensile Strength	(MPa)	54.9 ± 1.5	49.1 ± 1.3	42.7 ± 1.4	34.6 ± 1.5
90° Tensile Modulus	(GPa)	9.5 ± 0.3	9.0 ± 0.2	8.0 ± 0.4	7.8 ± 0.2
Flexural Strength	(MPa)	866.2 ± 12.7	810.4 ± 9.9	724.2 ± 11.2	664.3 ± 18.3
Flexural Modulus	(GPa)	62.0 ± 1.7	60.6 ± 1.5	57.3 ± 2.0	50.5 ± 1.6
Short-beam Shear Strength	(MPa)	73.8 ± 1.8	67.7 ± 1.9	58.6 ± 2.0	53.0 ± 2.0
Short-beam Shear Modulus	(GPa)	2.2 ± 0.06	2.0 ± 0.04	1.8 ± 0.05	1.5 ± 0.07

**Table 4:** Lifetime prediction of the CF/PMMA composite material.

Test Type	Strength Retention Rate	*d*
Tensile	80%	20.81
	50%	99.05
Shear	80%	27.48
	50%	389.06

## Data Availability

The original contributions presented in this study are included in the article. Further inquiries can be directed to the corresponding authors.
